# Effectiveness of treating depression with eye movement desensitization and reprocessing among inpatients–A follow-up study over 12 months

**DOI:** 10.3389/fpsyg.2022.937204

**Published:** 2022-08-10

**Authors:** Susanne Altmeyer, Leonie Wollersheim, Niclas Kilian-Hütten, Alexander Behnke, Arne Hofmann, Visal Tumani

**Affiliations:** ^1^Gezeitenhaus Traumahospital Schloss Eichholz, Wesseling, Germany; ^2^Clinical and Biological Psychology, Institute of Psychology and Education, Ulm University, Ulm, Germany; ^3^EMDR-Institute Germany, Gezeitenhaus Traumahospital Schloss Eichholz, Wesseling, Germany; ^4^Department of Psychiatry and Psychotherapy III, Ulm University, Ulm, Germany

**Keywords:** depression, EMDR, EMDR DeprEnd protocol, psychotherapy, follow up

## Abstract

Increasing prevalence of depression poses a huge challenge to the healthcare systems, and the success rates of current standard therapies are limited. While 30% of treated patients do not experience a full remission after treatment, more than 75% of patients suffer from recurrent depressive episodes. Eye Movement Desensitization and Reprocessing (EMDR) therapy represents an emerging treatment option of depression, and preliminary studies show promising effects with a probably higher remission rate when compared to control-therapies such as cognitive behavioral therapy. In the present study, 49 patients with severe depression were treated with an integrated systemic treatment approach including EMDR therapy that followed a specific protocol with a treatment algorithm for depression in a naturalistic hospital setting. Following their discharge from the hospital, the patients were followed up by a structured telephone interview after 3 and 12 months. 27 of the 49 (55%) patients fulfilled the Beck’s depression criteria of a full remission when they were discharged. At the follow-up interview, 12 months after discharge, 7 of the 27 patients (26%) reported a relapse, while the remaining 20 patients (74%) had stayed relapse-free. The findings of our observational study confirm reports of earlier studies in patients with depression, showing that EMDR therapy leads to a high rate of remission, and is associated with a decreased number of relapses. Patients with depression receiving EMDR treatment may be more resilient to stressors.

## Introduction

Eye Movement Desensitization and Reprocessing (EMDR) therapy was introduced in 1987 by Francine Shapiro as a treatment for post-traumatic stress disorder (PTSD). EMDR therapy has the goal to integrate maladaptive information into a functional memory network by focusing on stressful memory content and simultaneous bilateral stimulation (Shapiro, 2018). EMDR Therapy consists of a structured set of protocols and procedures based on the Adaptive Information Processing (AIP) Model. The AIP model postulates that traumatic experiences are stored in a dysfunctional and frozen state which enhances the possibility of suffering a mental disorder (Shapiro, 2018). A recent study of Baek et al. (2019) could demonstrate the plausible biological main mechanism of EMDR treatment by showing that a fear response learned through classical conditioning is unlearned more quickly if the mouse’s gaze or attention is directed back and forth with the aid of a moving LED light. Meanwhile, a large number of scientific studies are available that not only demonstrate the effect of EMDR in PTSD (Chen et al., 2014), but also that EMDR leads to symptom reductions in depressive disorders, anxiety disorders, and other medical conditions (Shapiro, 2018; Wilson et al., 2018).

Depression is one of the most common mental illnesses that pose major challenges to the healthcare system worldwide (suicides, work disability, inpatient treatment, and early retirement). With a lifetime prevalence of up to 20% (Ebmeier et al., 2006; Kessler and Bromet, 2013), depression is considered one of the most common mental disorders, affecting more than 350 million people worldwide (Depression, 2017). Current statistics also show that depression has become more prevalent due to the COVID-19 pandemic (Hawes et al., 2021). Depressive illness has been treated to date with behavioral therapy, psychodynamic psychotherapy and, in severe cases, additional medication (DGPPN, 2015). In severe forms of depression, patients must also be provided with inpatient treatment. However, the major problem is that many patients get readmitted as inpatients due to the high recurrence rate, i.e., after treatment, 30% of treated patients do not achieve a complete remission (cure) and more than 75% of patients suffer from recurrent depressive episodes (Maj et al., 1992; de Jong-Meyer et al., 2007; Fostick et al., 2010). Although psychotherapy and medication can improve remission rates, the success rates of currently available treatments are limited. Even after a successful treatment with full remission of symptoms, 40–50% of patients experience a relapse within the first year after completing treatment (Hollon et al., 1992; Hautzinger et al., 1996; de Jong-Meyer et al., 2007).

Stressful traumatic life events and stressful experiences (e.g., separation, loss, humiliation, embarrassment, serious illness, excessive demands, childhood abuse and maltreatment, neglect and other negative childhood stresses etc.) play a crucial role in the development of depression (Kendler et al., 2003; Teicher et al., 2006; Hase et al., 2018). Besides increasing the risk to develop depression, it also plays a crucial role concerning symptom severity and the course of depression (Karabatsiakis and Schönfeldt-Lecuona, 2020).

Eye Movement Desensitization and Reprocessing is an effective trauma focused approach to treat depression, and in clinical practice, since many years therapists use EMDR therapy to work with stressful and traumatic memories in depressed patients (Hofmann, 2020). Moreover, a recent systematic review and metanalysis by Carletto et al. (2021) and Yan et al. (2021) reported efficacy of EMDR therapy in the treatment of depression in 9 controlled studies. Most of these 9 studies show a higher rate of remission at the end of therapy compared to the control therapies (including cognitive behavioral therapy; Yan et al., 2021). Usually patients with full remissions of their depression have a significantly lower risk to relapse again (Paykel et al., 1995; Nierenberg et al., 2003). So far, no study has investigated long-term stability of EMDR therapy in treating depressive symptoms (Carletto et al., 2021). To advance the existing evidences, in our present study, the aim was to evaluate the efficiency and stability of treatment results with “EMDR therapy,” for depressive inpatients in the Gezeiten Haus, Hospital Schloss Eichholz.

## Materials and methods

The study aimed to show whether the intervention with EMDR leads to an improvement of symptoms in depressive patients and if the stability of the remission rates in patients treated with EMDR therapy are different from those mentioned in the previously reported literature. The protocol and implementation of the study was carried out according to the ethics committee Ärztekammer Nordrhein. The patients were selected according to their date of admission if they met the inclusion criteria.

**FIGURE 1:**
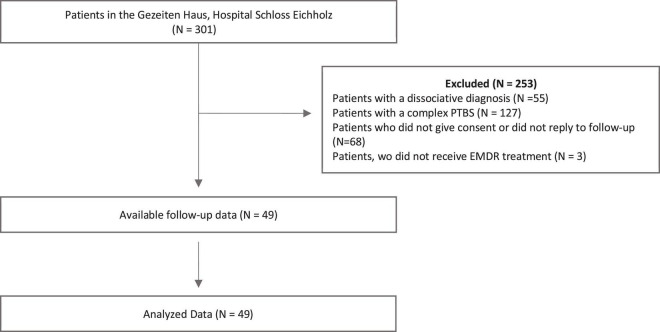
Overview of the data collection process.

Our study consisted of 49 inpatients who were treated in the Gezeiten Haus, hospital Schloss Eichholz (see Table 1 and Figure 1). The Gezeiten Haus hospital, Schloss Eichholz is a private hospital for psychotraumatology, EMDR, psychosomatics and traditional Chinese medicine. The focus of the specialist hospital is on integrative acute treatment of psychological and psychosomatic disorders, especially trauma related disorders and depression. The patients are usually referred to the hospital by psychiatrists and psychotherapists as well as other psychiatric units who recommend the clinic for a specialized intensive EMDR treatment because they don’t respond to guideline oriented treatment any more. The EMDR therapy followed the EMDR DeprEnd protocol and the eight-phase approach by Shapiro, 2018). The EMDR DeprEnd protocol is an EMDR treatment algorithm for depression that focuses on stressful or traumatic episode triggers of depressive episodes, negative belief systems, depressive or suicidal states (Hofmann et al., 2018).

**TABLE 1:** Sociodemographic characteristics of the study cohort (*n* = 49).

Gender (f/m)	32 (65.31%)/17 (34.69%)
Age (M (SD) (years)	46 (11.1)
**Marital status**	
In a relationship	9 (18.37%)
Married	24 (48.98%)
Divorced/separated	10 (20.41%)
Single	3 (6.12%)
No information	3 (6.12%)

The current study included all patients between 18 and 70 years of age with the diagnoses of depression (ICD 10 F32.XX, F33.XX) potentially with a combination of PTBS (ICD 10 F43.1). Exclusion criteria were acute suicidal tendencies, acute psychoses, manic episodes, severe dissociation, severe self-harm or addiction due to the additional complexity that these disorders add to the treatment time. Most patients (75.5%) were admitted with a prescribed medication. During hospitalization, the main focus is not on medication change, so patients are usually discharged with similar medications. If medications were administered, they had to be stable for 2–4 weeks for the patient to be enrolled in the study.

### Study design and procedure

As part of quality assurance, a series of diagnostic tests is routinely administered to all inpatients admitted to the Hospital Gezeiten Haus with written informed consent. This test package is given both at the beginning of the hospital stay and shortly before discharge.

The questionnaires cover the following topics: Symptomatic Distress in various domains [Symptom-Checklist (SCL-90-R)]; depressive symptoms [Beck Depression Inventory (BDI-II)]; Self-esteem [Rosenberg Self-esteem Scale (RSE)]; Dissociative phenomena [Dissociative Experiences Scale (DES)]; Trauma related disorder, intrusion, avoidance and hyperarousal [Impact of Events Scale (IES-R)]; childhood traumatic experiences [Childhood Trauma Questionnaire (CTQ)]; complex PTSD [Screening of complex post-traumatic stress disorder (Sk-PTBS)].

After the diagnostic phase, psychotherapeutic treatment is provided in an overall treatment plan, which includes psycho- and sociotherapeutic as well as psychoeducational and supportive measures in addition to medication. After a potential remission, BDI-II score under 12 (as per the BDI-II recommendation), of depressive symptoms and individual discharge preparations, patients were discharged. In order to examine the further course, especially after leaving the hospital, as follow-up, two telephone interviews were conducted both after 3 and 12 months. In the short telephone interview, experienced psychological staff asked the patients about their further therapeutic treatment after discharge, their current work situation, and whether they have had a relapse in the last 3 or 12 months. At the end of the telephone interviews, the former patients were asked to answer the three questionnaires. If they agreed, they were sent an e-mail with information and link for completion and submission of the questionnaires.

## Statistical analysis

All data were saved and analyzed in anonymized form using IBM SPSS Statistics (version 26.0.0) and R (version 4.1.3). Four-field tables on remission and relapse after therapy provided a descriptive overview of the success of treatment. Further analyses on the success of the treatment were performed using two-tailed paired *t*-tests for pre-post comparisons of symptom severity measures. Repeated measures ANOVAs with BDI-II and IES-R scores and overall symptom burden as dependent variable, measurement time (pre, post) as independent variable and the CTQ score as a potential covariate, as well as the interaction of time × CTQ score were conducted to determine a possible influence of childhood maltreatment on symptom reduction.

## Results

### Evaluation of treatment changes

**FIGURE 2:**
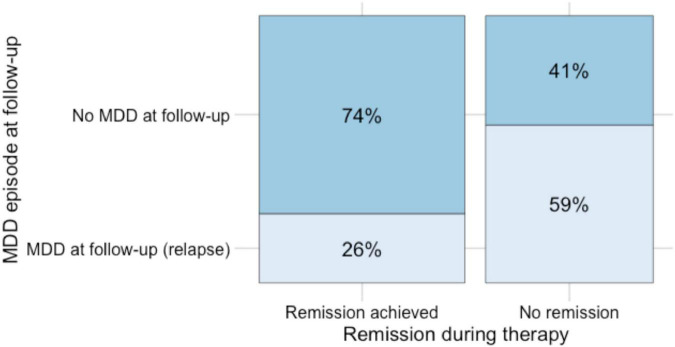
Distribution of relapse and remission in the study cohort.

A total of 49 of the patients who met the search criteria were analyzed. The average follow-up time at the end of the inpatient treatment was 13 and a half months (*MDN* = 11.5 months). Table 2 and Figure 2 display an overview of the distribution of remission and relapse from depressive symptoms in our study cohort. 27 (55.0%) of patients achieved a remission after therapy, 22 (44.9%) did not. If remission was achieved during therapy, 74.07% (20 out of 27) of cases remained stable during follow-up phase; 25.93% (7 out of 27) relapsed. If no remission was achieved during therapy, 59.09% (13 out of 22) relapsed and 40.91% (9 out of 22) achieved a remission during follow-up phase.

**TABLE 2:** Therapy-related characteristics of the study cohort (*n* = 49).

Number of EMDR sessions M (SD)	12.88 (6.02)
Remission (yes/no)	27 (55, 1%)/22 (44, 9%)
Relapse (yes/no)	20 (40, 82%)/29 (59, 18%)
Working (yes/no)	30 (63, 83%)/17 (36, 17%)

A total of 15 of the patients suffered from primary depression without PTSD comorbidity. Eleven of them suffered from recurrent depression, four suffered from a first episode of depression. In the follow-up (*MDN* = 12 months after discharge; *M* = 15 months after discharge) nine (60%) of these patients reported a remission from their depressive symptoms at the end of their inpatient therapy. After the 3-month follow-up one patient reported a relapse that occurred 3 months after being discharged from hospital. The others reported that they had not had a relapse at follow up (on average *M* = 12 months after discharge).

In comparison, in the group of patients with depression and comorbid PTSD, 18 (52.9%) patients had remissions at the end of treatment in the hospital and 12 of these patients also did not report relapse in the follow-up interview (*MDN* = 11 months after discharge; *M* = 13 months after discharge).

**FIGURE 3:**
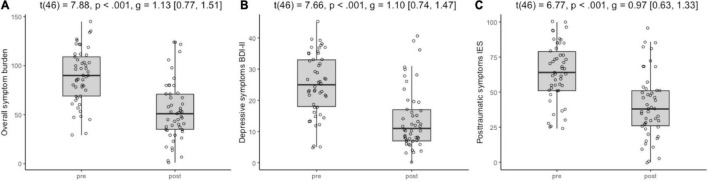
Symptom change from pre- to post Eye Movement Desensitization and Reprocessing (EMDR) treatment. **(A)** Overall symptom burden, **(B)** depressive symptoms BDI-II, **(C)** posttraumatic symptoms IES.

Paired *t*-tests were used to examine all pre- and post-treatment changes. Results revealed a statistically significant decline in depressive symptoms (*p* < 0.001; Figure 3B) and subjective distress caused by traumatic events (*p* < 0.001; Figure 3C) after treatment with EMDR. Similarly, overall symptom burden, as assessed by sum of depressive and distress symptoms, significantly decreased over time (*p* < 0.001; Figure 3A). All changes were considered very robust in effect (*g* > 1.0). Details of all pre-post comparisons can be seen in Figure 3.

**FIGURE 4:**
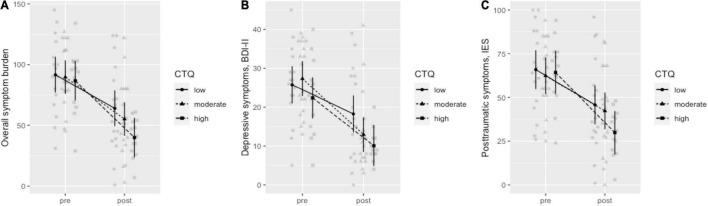
Influence of childhood maltreatment on therapy outcome. **(A)** Overall symptom burden, **(B)** depressive symptoms BDI-II, **(C)** posttraumatic symptoms IES.

A repeated measures ANOVA, *F*_(3, 60.4)_ = 24.6, *p* < 0.001, *R*^2^ = 0.289 (0.652), indicated that the reduction of overall symptom burden during therapy differed depending on the patients’ experience of child maltreatment (CM), Time × CM: *F*_(1, 45)_ = 3.14, *p* = 0.08, η^2^_*p*_ = 0.07. Explorative *post-hoc* tests showed that before therapy, the patients’ symptom burden did not correlate with their CM experiences, *d* = −0.03, *p* = 0.9. Through therapy, symptoms were reduced more in patients with more CM experiences (e.g., at CTQ score = 58: Cohen’s *d* = −1.94, *p* < 0.001) whereas symptom reduction was lower in patients with less CM experiences (e.g., at CTQ score = 32: Cohen’s *d* = −1.4, *p* < 0.001). Consequently, patients reporting more CM showed less symptom burden at post therapy, *d* = −0.46, *p* = 0.06. Results are visualized in Figure 4A (for statistical details, see Supplementary Tables 1a–c). Similar effect patterns could also be shown for the independent consideration of depressive symptoms (Figure 4B and Supplementary Tables 2a–c) and subjective distress caused by traumatic events (Figure 4C and Supplementary Tables 3a–c).

In this context, it was also investigated whether patients with depression as primary diagnoses differed from patients with depression and comorbid PTSD regarding their CM experiences. An independent *t*-test showed no significant difference, *t*(45) = 0.411, *p* = 0.683.

## Discussion

In a naturalistic setting, the present study investigated, whether EMDR intervention leads to a symptomatic improvement in depressed patients, and whether remission rates in EMDR-treated patients are stable at the follow-up (Hase et al., 2018; Ostacoli et al., 2018).

Randomized clinical trials have already shown that EMDR is an effective method for treating patients with depressive symptoms (Yan et al., 2021). In the present study comparable results could be obtained under naturalistic conditions. Ostacoli et al. (2018) reported a 71% remission rate in the EMDR group right after treatment and 54.8% stable remissions after 6 months follow-up. Hase et al. (2018) reported a 50% remission rate in patients with depression after being treated with EMDR therapy in an inpatient setting. Accordingly, in our study, we showed a comparable overall remission rate, although there was a difference in the remission rate between patients with depression as primary diagnosis and patients with a comorbid PTSD. Patients without PTSD had better remission rates after completion of EMDR therapy, which remained stable after an average of 1 year of follow-up in almost all cases. Here, it should be mentioned that in some previously listed studies, remission was considered to be a BDI-II value below 9. In our study, we defined remission as a BDI-II value below 12.

The results in our study population show fewer relapses (26% Figure 2) in depressed patients after successful treatment compared to relapse rates reported in previous literature (Hollon et al., 1992; Hautzinger et al., 1996; de Jong-Meyer et al., 2007). This finding is comparable to the follow-up results reported by Ostacoli et al. (2018), who reported 54.8% relapse free patients at 6 months follow-up in the group of depressed patients treated with EMDR.

Therefore, these results are consistent with recent EMDR studies reporting that EMDR therapy possibly leads not only to a higher rate of remissions in depressive patients, but also may decrease the number of relapses after treatment when compared to treatment as usual (Hase et al., 2018).

Childhood trauma is a major risk factor for developing depression (Edwards et al., 2003; Chapman et al., 2004; Humphreys et al., 2020). Adversities during childhood are not only associated with the severity (Rhebergen et al., 2012) and the chronicity (Nanni et al., 2012; Klein and Kotov, 2016) of the course of depression, but also with a longer time to remission (Fuller-Thomson et al., 2014). Heim et al. (2008) showed in their study that adverse childhood experiences and childhood trauma are associated with altered HPA axis potential as well as persistent sensitization of the stress response, which are also related to depressive symptoms. Studies show that neurobiological differences exist in depressed patients depending on whether trauma occurred in childhood or adolescence (Heim et al., 2004, 2008).

Heim et al. (2008) hypothesize that different therapeutic methods are required depending on whether childhood traumatization is present in depressed patients, with the aim to integrate the components of a neural network that have been altered by trauma, with the goal of normalizing neuroendocrine responsiveness and behavior. Notably, the present study also found that patients with more childhood trauma seem to have achieved better improvement during the hospital stay. In the light of the AIP model and regarding the results of the present study, this could mean that EMDR is particularly effective in patients belonging to the subgroup with childhood traumatization with corresponding biological changes.

Since a large number of studies show that emotional abuse and maltreatment are significantly more associated with depression compared to physical abuse (Mandelli et al., 2015; Humphreys et al., 2020), it would be relevant to investigate in future studies the effect of EMDR treatment in depressed patients, distinguished by the type of childhood maltreatment.

### Limitations

The present study has limitations that should be considered when interpreting the results. This is a naturalistic observational study, in which less factors could be controlled for. For example, medication change and additional treatments might have influenced the course of symptoms after discharge. Importantly in this regard, 91% of patients received an outpatient therapy after their hospital stay, and 25% of the patients were in the hospital during the COVID-19 pandemic which meant potential stress exposure within the hospital setting and after discharge. Another limitation of the present study is that the number of subjects (*N* = 49) was small. Further limitations include lack of randomization, and, more importantly, lack of a control group to compare the efficiency of EMDR with other depression-focused interventions. The reliability of the follow-up assessments may be lower due to use of telephone-based self-reporting instruments.

## Conclusion

In our study EMDR therapy leads to a high rate of remission and is associated with a decreased number of relapses in patients with depression. The results of the present observational study confirm data from previous research that EMDR is a promising method for treating depression as well as depressive symptoms in patients with history of childhood trauma. In the light of these results, future studies could further examine the effectiveness of EMDR treatment in patients with depressive disorders.

## Data availability statement

The original contributions presented in this study are included in the article/Supplementary material, further inquiries can be directed to the corresponding authors.

## Ethics statement

The studies involving human participants were reviewed and approved by the protocol and implementation of the study was carried out according to the ethics committee Ärztekammer Nordrhein. The patients/participants provided their written informed consent to participate in this study.

## Author contributions

AH and SA developed the study concept. LW and NK-H introduced and established the methodology. LW conducted the study setup, coordinated the study, and collected the psychological data. AB performed the statistical data analyses. VT supervised and coordinated the study. SA and LW drafted the manuscript under the supervision of AH and VT. All authors contributed to data interpretation, critically revised the manuscript, and approved its final version for submission.
