# Supplementary material for: Effectiveness of treating depression with eye movement desensitization and reprocessing among inpatients–A follow-up study over 12 months
Source: Front Psychol. 2022 Aug 10;13:937204. doi: 10.3389/fpsyg.2022.937204 (PMC9402253; doi:10.3389/fpsyg.2022.937204)
Supplement: Supplementary file 1 [file Data_Sheet_1.PDF]

Supplementary Table 1a

Results of repeated measures ANOVA analyzing the course of the overall symptom burden in patients

Model factors	*F*	*df*	*df.res*	*p*	*η²*
Time	70.09	1	45	< .001	.61
CTQ score	1.33	1	45	.26	.03
Time x CTQ score	3.14	1	45	.08	.07
Overall model statistic: $F(3, 60.4) = 24.6$, $p < .001$, $R^2 = .289$ (.652)					

Supplementary Table 1b

Post hoc tests on the association of CTQ scores with the overall symptom burden at each time point

Time	*b*	*SE*	*df*	*t*	*p*	*d*	*95% CI*
pre	-0.03	.25	71.4	-.12	.90	-.03	[-.49, .43]
post	-0.46	.25	71.4	-1.88	.06	-.44	[-.91, .03]

Supplementary Table 1c

Post hoc tests on the pre-post change in overall symptom burden at different CTQ values (e.g. 32, 46, 58)

CTQ score	*b*	*SE*	*df*	*t*	*p*	*d*	*95% CI*
32	28.7	5.73	45	5.02	<.001	1.40	[.80, 1.99]
46	34.7	4.27	45	8.14	<.001	1.69	[1.2, 2.17]
58	39.9	4.91	45	8.13	<.001	1.94	[1.38, 2.5]

Note: *The CTQ values 32, 46, and 58 represent the* M – 1SD, M, *and respectively* M + 1SD *of the distribution of reported experiences of childhood maltreatment.*

Supplementary Table 2a

Results of repeated measures ANOVA analyzing the course of depressive symptoms in patients

Model factors	*F*	*Df*	*Df.res*	*p*	*η²*
Time	67.04	1	45	< .001	.61
CTQ score	2.62	1	45	.11	.03
Time x CTQ score	2.95	1	45	.09	.07
Overall model statistic: $F(3, 60.4) = 23.9$, $p < .001$, $R^2 = .296$ (.646)					

Supplementary Table 2b

Post hoc tests on the association of CTQ scores with depressive symptoms at each time point

Time	*b*	*SE*	*df*	*t*	*p*	*d*	*95% CI*
pre	-0.04	.08	72.2	-.54	.59	-.13	[-.59, .34]
post	-0.18	.08	72.2	-2.26	.03	-.53	[-1, -.06]

Supplementary Table 2c

Post hoc tests on the pre-post change in depressive symptoms at different CTQ values (e.g. 32, 46, 58)

CTQ scores	*b*	*SE*	*df*	*t*	*p*	*d*	*95% CI*
32	9.29	1.89	45	4.92	<.001	1.37	[.78, 1.96]
46	11.22	1.41	45	7.96	<.001	1.65	[1.17, 2.13]
58	12.87	1.62	45	7.94	<.001	1.9	[1.34, 2.45]

Note: The CTQ values 32, 46, and 58 represent the *M* – 1*SD*, *M*, and respectively *M* + 1*SD* of the distribution of reported experiences of childhood maltreatment.

Supplementary Table 3a

Results of repeated measures ANOVA analyzing the course of posttraumatic symptoms in patients

Model factors	*F*	*df*	*df.res*	*p*	*η²*
Time	48.15	1	45	< .001	.61
CTQ score	0.71	1	45	.4	.03
Time x CTQ score	2.18	1	45	.15	.07
Overall model statistic: $F(3, 60.4) = 16.8$, $p < .001$, $R^2 = .239$ (.572)					

Supplementary Table 3b

Post hoc tests on the association of CTQ scores with posttraumatic symptoms at each time point

Time	*b*	*SE*	*df*	*t*	*p*	*d*	*95% CI*
pre	0.01	.19	75.6	.07	.95	.02	[-.44, 47]
post	-0.28	.19	75.6	-1.5	.14	-.34	[-.8, .11]

Supplementary Table 3c

Post hoc tests on the pre-post change in posttraumatic symptoms at different CTQ values (e.g. 32, 46, 58)

CTQ score	*b*	*SE*	*df*	*t*	*p*	*d*	*95% CI*
32	19.4	4.68	45	4.15	<.001	1.16	[.56, 1.74]
46	23.5	3.49	45	6.75	<.001	1.4	[.94, 1.86]
58	27.0	4.01	45	6.74	<.001	1.61	[1.08, 2.14]

Note: The CTQ values 32, 46, and 58 represent the $M - 1SD$, M, and respectively $M + 1SD$ of the distribution of reported experiences of childhood maltreatment.
